# Exploration of the Hungarian Version of Test Your Memory in General Practice: A Cross-Sectional Correlational Study of a Convenience Sample of Middle-Aged and Older Adults

**DOI:** 10.3390/geriatrics9030074

**Published:** 2024-06-03

**Authors:** Szabolcs Garbóczy, András Mohos, Anikó Égerházi, Anita Szemán-Nagy, János András Zsuffa, Szilvia Heim, Viktor Rekenyi, László Róbert Kolozsvári

**Affiliations:** 1 Department of Psychiatry, Faculty of Medicine, University of Debrecen, 4032 Debrecen, Hungary; 2 Doctoral School of Health Sciences, University of Debrecen, 4032 Debrecen, Hungary; 3 Department of Family Medicine, Faculty of Medicine, University of Szeged, 6725 Szeged, Hungary; 4 Department of Personality and Clinical Psychology, Institute of Psychology, University of Debrecen, 4032 Debrecen, Hungary; 5 Department of Family Medicine, Semmelweis University, 1085 Budapest, Hungary; 6 Neurocognitive Research Center, National Institute of Mental Health, Neurology and Neurosurgery, 1145 Budapest, Hungary; 7 Department of Primary Health Care, Medical School, University of Pécs, 7623 Pécs, Hungary; 8 Department of Family and Occupational Medicine, Faculty of Medicine, University of Debrecen, 4032 Debrecen, Hungary

**Keywords:** dementia, mild cognitive impairment, elderly, primary care, GP, family physician

## Abstract

Background: Dementia is a significant health issue worldwide. Mild cognitive impairment (MCI) can transform into dementia over time. General practitioners (GPs) may be the first to notice the cognitive deficit; therefore, it is crucial for them to have access to a screening test that can be administered quickly and efficiently. We explored the Hungarian version of the Test Your Memory self-administered dementia screening test in general practice for the early detection of dementia and cognitive impairment. Methods: In the four Hungarian cities with medical universities, 368 patients over the age of 50 attending GPs filled out the questionnaire within the framework of our cross-sectional study. Results: The total scores of the test showed a significant correlation with education and type of occupation and a significant negative correlation with age. The results of this research showed that the clock drawing and recall subtest scores deteriorate at the earliest age. Conclusion: The test can be filled out in a GP’s office easily and two of its subtests can raise the possibility that patients may need further assessment, especially if they have symptoms, at an earlier age than the other subtests.

## 1. Introduction

Due to the increasing burden of dementia on society in recent years, dementia research has become of paramount importance. It is essential to detect the early signs, diagnose the disease in time, and provide patients with the appropriate treatment as soon as possible [1]. Data show that the number of quality years a dementia patient can spend in a familial environment can be extended with therapy and early intervention [2,3]. With education, training, and support, the caregiver’s quality of life can also be improved [4]. This requires the education of the general population about the early signs of dementia in order to provide the public with the tools for widespread early prevention. Initiatives such as Alzheimer’s cafes and World Alzheimer’s Month have commenced and have been gaining popularity worldwide [5]. On the other hand, professionally prepared teams must wait for clients to apply for screening. Most people reluctantly go to the examination, and many are not even aware of the symptoms themselves; they usually complete the screening because of concerned loved ones. Considering the delicate nature of the symptoms—cognitive challenges that one may feel insecure, embarrassed, or defensive about—most patients prefer to reach out to their family doctor with dementia-related concerns because of their already-established rapport.

According to the international literature, the prevalence of mild cognitive impairment (MCI) in general practice is between 16 and 20%, while that of dementia is between 4 and 5.7% [6,7,8,9,10]. It is therefore of the utmost importance that family physicians have the appropriate tools at their disposal to diagnose the disease, especially once factors such as increased time pressure and the adequate distribution of available personal resources are taken into account as well. Although there are extensive screening tests available in Hungarian (The Alzheimer’s Disease Assessment Scale–Cognitive Subscale (ADAS-Cog), Montreal Cognitive Assessment (MoCA), and Addenbrooke’s Cognitive Examination (ACE)), these tend to be time-consuming and require professional personnel to administer them [11,12,13,14,15].

In Hungary, the screening of different types of dementia and the organizations focusing on dementia-centric patient care are both in their infancy. It is also not clear which profession should provide care for the clients. Although the provision and reception capacity of the social sphere has only just recently started to develop, the gaps and flaws of the system are obvious: private institutions provide their services at high prices while publicly funded institutions have serious performance limitations due to a lack of resources [16]. The general practitioner’s (GP) recommendation in Hungary does not include a screening protocol for the most common form of dementia, Alzheimer’s disease, only for the vascular type, and the application of it may also exceed the time and personnel resources available to the GP [17].

Examining the attitude of Hungarian GPs, researchers found that half of the specialists perform tests if cognitive decline is suspected and that, for them, time is the most crucial component of this entire process for various reasons: they want to have more time allocated for individual patient care and they also want to have access to the kind of effective, economically viable dementia detection tools that would save all involved parties valuable time. The other half of the specialists who did not perform any tests cited time constraints as the most common reason why they failed to assist their dementia patients in a more effective way [18,19]. Those family physicians who carry out dementia screening mainly use, in accordance with the current recommendations, the Mini Mental State Examination (MMSE) or the clock drawing test; however, both of these options are very time-consuming from the perspective of the medical personnel and are designed to detect signs of more severe types of dementia [20].

In our previous studies, due to the above reasons, we translated, examined, and validated a test that can be used in the screening of dementia that spares the doctor and their colleagues time-consuming test taking. This was Test Your Memory (TYM), the Hungarian version (TYM-HUN): a self-administered test that, by choosing the appropriate cut-off point, can be used very reliably for the screening of both early-stage dementia (sensitivity 94%, specificity 94%) and MCI (sensitivity 80%, specificity 96%) [21,22,23].

To the best of our knowledge, a comprehensive population screening with a dementia focus is yet to be carried out in Hungary. Concerning the epidemiology of the disease, conclusions were primarily drawn from the diagnoses that patients were given in the hospital and literature data [24,25]. There was a post-mortem study that found that the proportion of people suffering from vascular dementia in its sample was significantly higher than the international rate. However, the results obtained from this sample could not be interpreted for the entire Hungarian population [26]. Perhaps this was also due to a Hungarian phenomenon where, according to a 2019 study, GPs deemed the most common form of this disease in Hungary to be the vascular type of dementia. This could be the primary reason why an officially recommended screening protocol is only available for this type of dementia on the Hungarian General Practice Partnership’s website; however, this could also be the result of the distorted collective opinion of GPs [27].

## 2. Materials and Methods

### 2.1. Participants and Location

Employing the TYM-HUN test, we wanted to assess the cognitive abilities of the population over 50 in the general practice of the four Hungarian cities with medical universities. Utilizing the answers of the respondents, we tried to find correlations between the demographic data and the scores of the TYM test. To do so, we disseminated TYM-HUN in the four Hungarian cities with medical universities: Budapest, Pécs, Szeged, and Debrecen. The participants completed the tests between 15 June and 15 December 2022. We put together a test battery for family doctors willing to cooperate in these Hungarian cities which included written consent, a demographic data survey, and the TYM-HUN test itself. The demographic questions evolved and, after we expanded them, included education, marital status, number of people living in the same household, active/retired lifestyle, type of occupation, regular exercise, depressed mood, and forgetfulness experienced by self or a relative/professional. We planned to take a total of 400 tests from the four cities; therefore, we disseminated 400 tests. We received a total of 368 fully completed tests from the family doctors: 9 respondents did not fall within the age criteria, while 23 changed their mind while completing the test and did not complete the test or did not sign their consent to participate in the study. Therefore, we can say that convenience sampling took place, which was carried out by family doctors who were motivated to participate in the study, for their patients who also had the desire to take part in the examination.

### 2.2. The Study Instrument

The TYM test is a paper-based, two-sided test that is self-administered. The first page starts with questions about orientation: name, date of birth, age, and date of completion (10 points), followed by sentence copying (2 points) and a reminder of the sentence. After that, there is a test of current and past general knowledge (3 points) and then simple calculations (4 points) and a verbal fluency test (4 points). The next step is identifying similarities (4 points). At the bottom of the page, they receive a second reminder about the copied sentence. On the second page, they have to recognize and name the parts of a figure (5 points) and then connect the dots forming a letter, testing visuospatial skills (3 points). This is followed by a clock-drawing test (4 points) and, finally, the previously copied sentence, for which they were given two reminders that must be recalled (6 points). At the end of the test, 5 points are added for how much help the person completing the test had to be provided; the less help they receive, the more points are added [21,22]. From the details above, it can be seen that the TYM is a very complex test that examines many cognitive areas.

When performing the tests, we asked colleagues to provide minimal supervision. It also proved useful when a relative could accompany the patient and someone could supervise the process, during which, the most important rule was that the test taker could not turn the sheet back once it had been turned over. The delayed recall of the sentence is the subtask that is the most sensitive to Alzheimer’s dementia and amnestic MCI [28,29,30]. Due to the content of the test (questions about date of birth and current date), we asked the colleagues who completed the tests in their practices to evaluate the test responses as well.

### 2.3. Aim of the Study

From the results of the tests, we tried to determine which demographic characteristic the TYM-Hun score correlated with. In addition, we also divided the test into its subtests to determine which subtest deteriorated at the earliest age. We hypothesized, as we found in our previous studies, that there would be no correlation between education, gender, or mood and the test score. However, we hypothesized that those who lived alone and those who led a sedentary lifestyle would achieve a lower score.

### 2.4. Statistics

We analyzed our data by using SPSS (v.26) and presented them as means, medians, and frequencies. Since the variables had non-normal distribution, we used Mann–Whitney U tests, Kruskall–Wallis tests, and Spearman rank correlations to compare the scores of groups and examine the relationship between the factors. To investigate in which age group a decreased score in different cognitive functions appears first, we performed Chi-square tests, and when the cell frequency was less than 5, we used Fisher’s exact test.

The variables of TYM were hardly skewed. We used Spearman correlations initially to examine the association between age and cognitive performance across all participants, treating age as a continuous variable. By employing Spearman correlations, we were able to explore the overall trend or pattern of association between age and cognitive performance, without imposing assumptions about the linearity of the relationship. This initial analysis provided insight into the general relationship between age and cognitive functioning in our sample.

Following this initial exploration, we then grouped age into categories to further investigate potential differences in cognitive performance across distinct age groups. This approach allowed for a more detailed examination of age-related trends in cognitive functioning, complementing the initial Spearman correlation analysis. Furthermore, we grouped the subtests into perfect and not-perfect scores to address the challenge of ceiling effects, where a significant proportion of participants achieved scores near the maximum. The other reason for this was that there is no Hungarian adaptation of the test to determine performance levels. By categorizing participants based on their performance, we aimed to mitigate the impact of ceiling effects and enable a more nuanced analysis of cognitive abilities within the sample. This approach facilitated the exploration of potential differences in cognitive functioning between individuals demonstrating exceptional proficiency and those who did not, enhancing the interpretability of our findings.

### 2.5. Ethical Approval

All patients gave written informed consent to participate in this study. We received permission from the Hungarian Medical Research Council’s Scientific and Research Ethics Committee to conduct our study, with permission number 28507-2/2019/EKU.

## 3. Results

### 3.1. Descriptive Data

In total, 368 participants filled out our questionnaires with an average age of 69.71 years and a median age of 70. Table 1 shows the demographic characteristics of our research. We also included participants with missing data because, additionally, after our data collection process had commenced, we added more demographic factors to be considered in our analysis and were not able to collect these data from the previously surveyed participants. This allowed us to gain a more comprehensive understanding of the relationship between demographic factors and cognitive function scores. We changed the study design because we felt that we could expand the range of interpretability and correlations if we asked a wider, more comprehensive range of demographic questions, which can presumably be related to cognitive decline on some level. Initially, only questions about age, gender, family status, number of children, and self-assessed mood were included in the questionnaire; then, after 112 questionnaires were collected, we added to these the number of people living in the same household, type of occupation, physical activity, and the presence of perceived memory problems for the demographic questions.

Table 2 shows the categories we determined in our previous studies: scoring 50–45 points on the TYM-Hun suggests a mentally healthy individual; scoring between 44 and 36 raises the possibility of MCI; and a score of 35 or less can indicate that further assessment is needed in the direction of dementia, especially if the respondent has symptoms [22,23].

### 3.2. Results of the Correlation of Subtest and Total Test Scores with Demographic Characteristics

Table 3 shows both the medians and means for the scores because, in some cases, where the median scores were the same across groups, reporting the means helped to show any subtle differences in the outcomes. The results of our study show that there were significant differences in cognitive function scores based on the level of education. Individuals with higher levels of education scored better in the cognitive function domains of Copying, Facts, Sums, Similarities, Visuospatial skills, Clock drawing, Recall, and Help and in total compared to those with lower levels of education. In regard to occupation, participants who were retired reported lower levels of cognitive function in the domains of Similarities, Visuospatial skills, Recall, and Help and in the total score than participants who were still professionally active. Physical workers had lower scores regarding Recall, while passive and active mental workers scored higher. Participants who were not doing sports for 3 × 45 min/week had weaker arithmetic skills. Subjects who reported feeling a depressed mood had poorer performance in the areas of Orientation, Copying, Visuospatial skills, and Recall. Individuals who acknowledged a memory problem showed a decline in their scores in the domain of Recall and in their total scores. Participants whose memory issues were recognized by others reported lower levels of cognitive function concerning Fluencies and Recall and obtained lower total scores.

We examined the correlation between cognitive function and demographic factors using the TYM test. We found that age had a weak, significant negative correlation with the domains of Copying, Facts, Sums, Similarities, Naming, Visuospatial skills, Clock drawing, Recall, and Help, as well as a moderate, significant negative correlation with the total score. Additionally, we found that the number of children had no significant correlation with any of the cognitive function domains, and the number of persons in the same household had a weak, significant positive correlation with the domains of Recall and Help (Table 4).

### 3.3. Results of Lower-Than-Expected Scores on Each Subtest in Different Age Groups

Table 5 shows the count and expected count of individuals in the 50–65, 66–80, and 80+ age groups who scored less than the maximum on various domains of the TYM test. The chi-square test results indicate that there was a statistically significant difference between the age groups and the scores of different cognitive abilities. The oldest group scored lower in the Copying, Similarities, and Visuospatial skills domains (*p* < 0.001, *p* = 0.001, *p* < 0.001), while the Clock drawing and Recall areas showed a deficit in the age group of 66–75 years, with higher observed counts than expected in the groups with lower scores (*p* < 0.001, *p* < 0.001).

## 4. Discussion

The results of our study conducted in four university cities illustrate that a non-negligible part of the participants visiting a family doctor without a diagnosis of dementia displayed symptoms of cognitive difficulties. According to our previous studies and the current test results, 32.6% of a GP’s patients over 50 may need to be further evaluated, especially if they have symptoms of cognitive decline. 

The trend is that some MCIs transform into dementia, while others do not [31,32,33]. If we take the cases we identified as needing further evaluation as a foundation and estimate the prevalence of MCI and dementia by using international trends, we obtain concerning results for the future that require immediate, or at least urgent, vigorous steps at the national level, with a special focus on strengthening the social sphere by legislators and policy-makers.

Nearly 40% of Hungary’s population is over 50 years of age (3,863,828 people) [34]; therefore, in the future, dementia-related health struggles are expected to put a significant strain on the Hungarian health sector, which is already under pressure due to a significant number of people living with dementia at this time who receive in-patient care only; a considerably lower percentage of them face struggles stemming from dementia in the social sphere, which is an even more disadvantaged position. Hospitalization is the most expensive form of dementia care [35]. Considering the mental burden this care comes with, it also very often leads to the death of the admitted client. Unfortunately, in Hungary, the most common case is that people with dementia die in hospital [36,37]. It is also not uncommon that at the time of the discovery of dementia, the person is admitted to a hospital ward; this could be prevented if patients were screened for dementia in GP practices. A high percentage of dementia patients could also be treated with intensive outpatient care, but, instead, they are admitted (unnecessarily) to the hospital [38].

According to recent surveys, we spend only one-fifth of the European average on financing health services for people with dementia, and the number of long-term care institutions is also very limited and inaccessible due to high costs. Another significant obstacle is that the services provided by the state do not allow family members to care for their loved ones, the people living with dementia, at home [16,39]. We believe that the most manageable and cost-effective way to intervene in this overload is at the level of secondary prevention, for which the application of TYM-HUN is perfectly suitable, a test that we strongly recommend to GPs for screening for cognitive disorders.

Not all of our hypotheses about TYM-HUN could be corroborated. In our current study, those who had lower educational levels performed worse compared to those who had degrees. Implementing targeted educational programs for families can reduce their burdens and depressive symptoms caused by uncertainty about handling their family member’s condition. Educating patients can also be a possible treatment to reduce their general behavioral problems and might delay their institutionalization. [40,41].

Gender and self-reported mood were not significantly correlated with the total scores as we presumed they would be. However, it was also not possible to support the notion that self-reported exercise can effectively delay the decrease in TYM-HUN total scores. Finally, the results partially supported our hypothesis that the more people living in the household, the more delayed the cognitive decline is.

Dividing the test into its subtests and analyzing them accordingly, taking into account different demographic characteristics, allowed us to determine in which subgroups which subtests showed impairment. Several demographic characteristics showed differences in distinct areas, but, overall, the total score of the test was significantly influenced by only four parameters: education, type of occupation, and memory problems reported by oneself or others. This means that the test, as it examines several cognitive domains, gives an excellent picture of the cognitive abilities of the person completing it, and, although there may be differences in several subtests depending on demographic characteristics, the total score of the test is affected significantly only by a few factors. Even these few are already known to modify cognitive abilities in a negative direction, such as education or retirement, e.g., use it or lose it [42,43,44]. Also, the memory problems reported by oneself or by others lead to the conclusion that whenever an individual is struggling with such issues or someone else notices them struggling in this context, direct screening for dementia is recommended [45,46].

On a practical level, the most important result of our research is that we found which two subtests show a difference at the earliest age: drawing a clock and recalling sentences. From this point of view, our advice to GPs has proven to be useful, i.e., to carefully supervise so that the person taking the test cannot turn their paper back over once it has already been turned to the other side. In this sense, the national recommendation, which we referred to earlier, can be relevant and can help in early detection as it contains exactly these two elements. However, we are still convinced that compared to the recommended tests, TYM-HUN is a test that can be taken using fewer human resources; it also requires minimal supervision and examines numerous cognitive areas in more detail.

Based on these two subtests, or even the entire TYM test, it would be worthwhile to create an online version that could prove to be even more accessible for patients and doctors alike. This would be beneficial for the following reasons: it could easily eliminate the possibility of fallacies; the time of administration could be measured, which could serve as an additional clue towards diagnosis; the older age group is also quite good with technology and most of them are expected to have the ability to complete such a test; it could also be shared on social media platforms that the elderly population often uses; due to anonymity, participation would also become largely risk-free, so the stressful effect of the doctor’s office would be negated and the fear of the stigma associated with the diagnosis could be reduced; and participants would receive immediate feedback elicited from the score achieved [47].

Reducing functional decline in the early stages of dementia is one of the most important tasks to mention in primary care. A study conducted in Hong Kong found that combining cognitive training, mind–body physical exercise, and nurse-led risk factor modification in a multi-domain approach might be applicable and may reduce the cognitive decline of elderly patients who have MCI. Although the study was a pilot, it raised some cardinal questions concerning the topic [48]. A working group of national experts also developed new recommendations feasible for primary care clinicians to initiate primary prevention conversations about cognitive decline [49]. Memory clinics might also be a great help in close working relationships with primary care [50].

## 5. Strengths and Limitations

The easy applicability of this test is supported by the fact that the gathering of the data was conducted during the time of COVID-19 and, despite the measurements and restrictions ordered because of the pandemic, the GPs were still able to recruit the participants and the patients were able to fill out our tests. Although, the medical personnel time and surveillance needed also decreased. So while the tool was self-administered, some participants may have received more support in filling out the questionnaire than others. The generalizability of the results of our survey has serious limitations: we used convenience sampling; the tests were completed only in urban and not in rural regions; and motivated GPs performed the tests on motivated patients. Although we were not able to reach a representative sample, we believe that we managed to achieve valuable results and a relatively high sample number with the screening test carried out in Hungarian GP practices. These results can also be considered a pilot study for a later, comprehensive, and representative survey.

Although we did not collect the opinions of the GPs who participated in this study in writing, they expressed their opinions about the test orally. Summarizing their subjective opinions, the GPs reported that giving feedback about the test result could help to involve the patient in the study. They added their practical observations that because older age can often lead to a loss of visual acuity, and since TYM-HUN is self-administered, it is recommended that the patient bring glasses to the office. The GPs recommended minimal surveillance, for example, using a clipboard when filling out the test, and for staff to turn the sheet over to prevent the patient from turning the page back. They found the test to be sufficiently sensitive as it examines several subdomains.

## 6. Conclusions

Our study may present a cause for concern, especially if we factor in the number of cognitively challenged people currently living at home who may have not yet been diagnosed. This calls for a comprehensive investigation and quick action at the appropriate levels; otherwise, the situation threatens to head towards a healthcare crisis once we factor in that in many places in Hungary, the care of people living with dementia for a longer period of time is currently assigned to hospitals instead of the appropriate social sector.

Based on our study and verbal feedback from GPs, the TYM-HUN seems to be a viable and user- and staff-friendly screening test that can reveal needs for further assessment in the direction of MCI and dementia in general practice. Therefore, we recommend the test for everyday use in the case of people who report or show signs of possible cognitive decline and also to keep in mind these family doctors’ observations during use.

## Figures and Tables

**Table 1:** Demographic characteristics.

	N	Missing
Gender		
Female	223	0
Male	145	
Age groups		
≤65	116	0
66–75	147	
≥76	105	
Education		
Primary education or less	103	1
Secondary education	153	
Bachelor’s or higher	111	
Marital status		
In a relationship	225	1
Divorced	55	
Single	24	
Widowed	63	
Number of persons living in the same household		
0	73	118
1	111	
2	40	
3	12	
4 or more	14	
Occupation		
Active worker	42	113
Retired	213	
Type of occupation		
Passive mental work	63	135
Active mental work	82	
Physical work	88	
Doing sports for 3 × 45 min/week		
Yes	85	117
No	166	
Depressed mood		
Yes	51	5
No	312	
Self-detected memory problems		
Yes	137	114
No	117	
Memory problems detected by others		
Yes	45	112
No	211	
Number of children		
0	5	256
1	26	
2	60	
3 or more	21	

**Table 2:** Frequency of 50–45, 44–36, and 35 or lower scores on the TYM-HUN.

TYM-HUN Scores	Number of Cases	Percentage
50–45	248	67.4
44–36	93	25.3
35 or less	27	7.3
Total	368	100

**Table 3:** Subtest medians and means according to the different demographic characteristics.

Demographic Factors	Orientation		Copying		Facts		Sums		Fluencies		Similarities		Naming		Visuospatial Skills		Clock Drawing		Recall		Help		Total	
	*	**	*	**	*	**	*	**	*	**	*	**	*	**	*	**	*	**	*	**	*	**	*	**
Gender																								
Female	10	9.65	2	1.83	3	2.63	4	3.61	4	3.68	4	6.62	5	4.8	3	2.46	4	3.35	6	4.78	5	4.64	47	45.04
Male	10	10	2	1.84	3	2.7	4	3.48	4	3.69	4	3.55	5	4.7	3	2.57	4	3.36	5	4.36	5	4.61	46	44.42
*p*-value	0.321		0.976		0.105		0.137		0.772		0.478		0.131		0.450		0.682		0.106		0.653		0.120	
Education																								
Primary education or less	10	9.48	2	1.68	3	2.45	4	3.27	4	3.5	4	3.28	5	4.67	3	2.21	4	3.16	5	3.79	5	4.39	44	41.87
Secondary education	10	9.71	2	1.91	3	2.66	4	3.64	4	3.71	4	3.71	5	4.85	3	2.54	4	3.47	6	4.9	5	4.69	47	45.77
Bachelor’s or higher	10	9.66	2	1.87	3	2.86	4	3.71	4	3.81	4	3.71	5	4.72	3	2.68	4	3.38	6	4.98	5	4.77	47	46.14
*p*-value	0.116		0.000		0.000		0.003		0.202		0.002		0.150		0.001		0.031		0.000		0.013		0.000	
Marital status																								
In a relationship	10	9.59	2	1.86	3	2.71	4	3.52	4	3.65	4	3.61	5	4.78	3	2.56	4	2.42	6	4.75	5	4.7	47	45.14
Divorced	10	9.71	2	1.85	3	2.55	4	3.64	4	3.71	4	3.58	5	4.78	3	2.4	4	3.4	6	4.67	5	4.6	47	44.89
Single	10	9.83	2	1.79	3	2.75	4	3.71	4	3.71	4	3.58	5	4.5	3	2.25	3	2.88	5	4.13	5	4.58	46	43.71
Widowed	10	9.65	2	1.78	3	2.59	4	3.6	4	3.75	4	3.54	5	4.78	3	2.43	4	3.25	5	4.29	5	4.44	45	44.1
*p*-value	0.289		0.512		0.132		0.896		0.657		0.820		0.071		0.242		0.054		0.241		0.080		0.108	
Occupation																								
Active worker	10	9.64	2	1.93	3	2.76	4	3.67	4	3.79	4	3.83	5	4.76	3	2.88	4	3.67	6	5.38	5	4.9	48,5	47.21
Retired	10	9.57	2	1.77	3	2.58	4	3.49	4	3.67	4	3.6	5	4.69	3	2.38	4	3.23	5	4.49	5	4.5	46	43.97
*p*-value	0.808		0.095		0.110		0.302		0.711		0.019		0.721		0.003		0.018		0.004		0.005		0.000	
Type of occupation																								
Passive mental work	10	9.63	2	1.87	3	2.71	4	3.78	4	3.7	4	3.75	5	4.84	3	2.6	4	3.33	6	5.03	5	4.52	47	45.78
Active mental work	10	9.6	2	1.84	3	2.66	4	3.62	4	3.79	4	3.74	5	4.71	3	2.56	4	3.41	6	4.96	5	4.76	47	45.66
Physical work	10	9.53	2	1.69	3	2.51	4	3.31	4	3.52	4	3.44	5	4.59	3	2.31	4	3.19	5	4.05	5	4.49	45	42.64
*p*-value	0.571		0.073		0.084		0.005		0.200		0.081		0.496		0.165		0.350		0.006		0.394		0.106	
Doing sports for 3 × 45 min/week																								
Yes	10	9.66	2	1.82	3	2.58	4	3.35	4	3.71	4	3.67	5	4.62	3	2.48	4	3.27	6	4.76	5	4.72	47	44.65
No	10	9.55	2	1.8	3	2.65	4	3.61	4	3.67	4	3.61	5	4.74	3	2.46	4	3.3	5	4.58	5	4.51	47	44.48
*p*-value	0.316		0.571		0.278		0.039		0.980		0.871		0.391		0.785		0.548		0.253		0.126		0.495	
Depressed mood																								
Yes	10	9.27	2	1.65	3	2.53	4	3.41	4	3.59	4	3.61	5	4.76	3	2.24	4	3.31	5	4.33	5	4.49	45	43.2
No	10	9.71	2	1.88	3	2.68	4	3.58	4	3.7	4	3.58	5	4.76	3	2.55	4	3.36	6	4.67	5	4.65	47	45.11
*p*-value	<0.001		0.003		0.127		0.119		0.383		0.483		0.737		0.045		0.410		0.026		0.408		0.038	
Self-detected memory problems																								
Yes	10	9.51	2	1.79	3	2.57	4	3.55	4	3.65	4	3.62	5	4.63	3	2.37	4	3.34	5	4.47	5	4.52	46	44.01
No	10	9.68	2	1.82	3	2.67	4	3.49	4	3.74	4	3.66	5	4.79	3	2.57	4	3.26	6	4.87	5	4.62	47	45.17
*p*-value	0.144		0.424		0.344		0.598		0.433		0.514		0.332		0.186		0.914		0.043		0.902		0.022	
Memory problems detected by others																								
Yes	10	9.42	2	1.6	3	2.47	4	3.64	4	3.47	4	3.71	5	4.47	3	2.24	4	3.16	5	3.53	5	4.31	45	42.02
No	10	9.62	2	1.84	3	2.64	4	3.5	4	3.73	4	3.62	5	4.75	3	2.51	4	3.33	6	4.88	5	4.63	47	45.05
*p*-value	0.805		0.060		0.150		0.470		0.037		0.546		0.103		0.314		0.407		<0.001		0.143		0.017	

* median. ** mean.

**Table 4:** The effect of age, children, and household members on the TYM-HUN scores.

Demographic Factors	Orientation	Copying	Facts	Sums	Fluencies	Similarities	Naming	Visuospatial Skills	Clock Drawing	Recall	Help	Total Score
Age	−0.089	−0.221 **	−0.133 *	−0.116 *	−0.530	−0.161 *	−0.148 *	−0.260 **	−0.268 **	−0.269 **	−0.322 **	−0.401 **
Number of children	0.030	0.008	0.017	−0.121	0.033	−0.165	0.175	0.092	−0.035	0.092	−0.145	0.012
Number of persons in the same household	−0.066	0.034	−0.030	0.047	−0.022	−0.005	−0.079	0.071	0.048	0.128 *	0.166 *	0.121

* *p* < 0.05. ** *p* < 0.01.

**Table 5:** Chi-square tests on the different subtests in three age categories.

Subtest		Age (Years)				
Orientation		≤65	66–75	76≤	Total	Chi-Square Test
10 points	Count	90	111	72	273	χ2(2) = 2.563; *p* = 0.278
	Expected Count	86.1	109.1	77.9		
Less than 10 points	Count	26	36	33	95	
	Expected Count	29.9	37.9	27.1		
	Total	116	147	105	368	
Copying		Age (years)				
		≤65	66–75	76≤	Total	Fisher’s Exact Test
2 points	Count	112	132	84	328	p(2) = 15.687; *p* < 0.001
	Expected Count	103.4	131	93.6		
Less than 2 points	Count	4	15	21	40	
	Expected Count	12.6	16	11.4		
	Total	116	147	105	368	
Facts		Age (years)				
		≤65	66–75	76≤	Total	Chi-Square Test
3 points	Count	89	103	66	258	χ2(2) = 5.057; *p* = 0.080
	Expected Count	81.3	103.1	73.6		
Less than 3 points	Count	27	44	39	110	
	Expected Count	34.7	43.9	31.4		
	Total	116	147	105	368	
Sums		Age (years)				
		≤65	66–75	76≤	Total	Chi-Square Test
4 points	Count	91	100	68	259	χ2(2) = 5.602; *p* = 0.061
	Expected Count	81.6	103.5	73.9		
Less than 4 points	Count	25	47	37	109	
	Expected Count	34.4	43.5	31.1		
	Total	116	147	105	368	
Fluencies		Age (years)				
		≤65	66–75	76≤	Total	Chi-Square Test
4 points	Count	93	117	81	291	χ2(2) = 0.345; *p* = 0.842
	Expected Count	91.7	116.2	83		
Less than 4 points	Count	23	30	24	77	
	Expected Count	24.3	30.8	22		
	Total	116	147	105	368	
Similarities		Age (years)				
		≤65	66–75	76≤	Total	Chi-Square Test
4 points	Count	96	114	65	275	χ2(2) = 13.724; *p* = 0.001
	Expected Count	86.7	109.9	78.5		
Less than 4 points	Count	20	33	40	93	
	Expected Count	29.3	37.1	26.5		
	Total	116	147	105	368	
Naming		Age (years)				
		≤65	66–75	76≤	Total	Chi-Square Test
5 points	Count	105	122	83	310	χ2(2) = 5.747; *p* = 0.056
	Expected Count	97.7	123.8	88.5		
Less than 5 points	Count	11	25	22	58	
	Expected Count	18.3	23.2	16.5		
	Total	116	147	105	368	
Visuospatial Skills		Age (years)				
		≤65	66–75	76≤	Total	Chi-Square Test
3 points	Count	104	113	64	281	χ2(2) = 25.188; *p* < 0.001
	Expected Count	88.6	112.2	80.2		
Less than 3 points	Count	12	34	41	87	
	Expected Count	27.4	34.8	24.8		
	Total	116	147	105	368	
Clock Drawing		Age (years)				
		≤65	66–75	76≤	Total	Chi-Square Test
4 points	Count	91	89	49	229	χ2(2) = 23.979; *p* < 0.001
	Expected Count	72.2	91.5	65.3		
Less than 4 points	Count	25	58	56	139	
	Expected Count	43.8	55.5	39.7		
	Total	116	147	105	368	
Recall		Age (years)				
		≤65	66–75	76≤	Total	Chi-Square Test
6 points	Count	74	74	39	187	χ2(2) = 15.684; *p* < 0.001
	Expected Count	58.9	74.7	53.4		
Less than 6 points	Count	42	73	66	181	
	Expected Count	57.1	72.3	51.6		
	Total	116	147	105	368	
Help		Age (years)				
		≤65	66–75	76≤	Total	Chi-Square Test
5 points	Count	108	116	63	287	χ2(2) = 35.304; *p* < 0.001
	Expected Count	90.5	114.6	81.9		
Less than 5 points	Count	8	31	42	81	
	Expected Count	25.5	32.4	23.1		
	Total	116	147	105	368	

## Data Availability

The data presented in this study are available upon request from the corresponding author. The data are not publicly available due to ethical reasons.
